# Prevention and Treatment of Traumatic Brain Injury Due to Rapid-Onset Natural Disasters

**DOI:** 10.3389/fpubh.2014.00028

**Published:** 2014-04-14

**Authors:** James L. Regens, Nick Mould

**Affiliations:** ^1^OUHSC Center for Biosecurity Research, University of Oklahoma Health Sciences Center, Oklahoma City, OK, USA

**Keywords:** traumatic brain injury, rapid-onset natural disasters, prevention, treatment

## Abstract

The prevention and treatment of traumatic brain injury (TBI) attributable to rapid-onset natural disasters is a major challenge confronting disaster preparedness planners and emergency medical personnel responding to those incidents. The kinetic energy released by rapid-onset natural disasters such as earthquakes, hurricanes or typhoons, and tornadoes can cause mild, moderate, or severe TBIs. As a result, neurotrauma is a major risk factor for mortality and morbidity outcomes within the spatial domain impacted by a rapid-onset natural disaster. This review article elucidates major challenges associated with immediate emergency medical response, long-term care, and prevention of post-event increases in pediatric TBIs because of child abuse when rapid-onset natural disasters occur.

## Introduction

Although the frequency and severity of rapid-onset natural disasters varies spatially and temporally, a review of the historical record demonstrates that earthquakes, floods, hurricanes or typhoons, tornadoes, and tsunamis are not rare events globally. For example, the WHO collaborating centre for research on the epidemiology of disasters (CRED) maintains an emergency events database (EM-DAT) that contains information about the occurrence and effects of more than 18,000 mass disasters that have happen in the world since 1900. A search of the EM-DAT reveals that the total number of rapid-onset natural disasters and slow-onset crises – drought and armed conflict – reported each year that either kill ≥10 people or leave ≥100 people injured, homeless, displaced, or evacuated as well as events that result in a country formally declaring a natural disaster and/or requesting international assistance reported each year has been steadily increasing in recent decades (1). For example, tropical cyclones are estimated to have caused 1,330,000 deaths between 1900 and 2014 (2). Within that overall time period, approximately 1,080 tropical cyclones occurred between 1980 and 2014, resulting in 412,644 deaths and 290,654 injuries (2). In part, the change in deaths and injuries reported reflects patterns of population migration to as well as population density in disaster-prone areas within developing countries in Africa, Asia, and Oceania. Developed countries also have experienced significant damage due to rapid-onset natural disasters (3). This is illustrated by the fact that in the United States (US) there were approximately 58,169 tornadoes between 1950 and 2014 that resulted in 6,697 deaths and 104,597 injuries (4). On a global scale, there were 125 earthquakes that are known to have caused more than 1,000 deaths per event between 1900 and 2014. The total number of deaths caused by these 125 major earthquakes is estimated to be 2,309,716 (5).

However, several caveats are warranted before one automatically concludes that the frequency of rapid-onset natural disasters is increasing. First, the overall size of the human population has been increasing at an exponential rate. With more people, vulnerability to deaths and injuries from rapid-onset natural disasters increases because there are more people to be affected by otherwise natural events. For example, the number of people living in coastal areas has grown dramatically. These low-lying areas are vulnerable to natural hazards such as tropical cyclones, tsunamis, floods, and, to some extent, earthquakes. Second, our ability to communicate news of both rapid-onset natural disasters as well as slow-onset crises has been increasing, especially since the invention of the internet and social media. It is possible that earlier in human history there may have been similar trends and variability with respect to natural disasters, but there were few ways the news of such disasters could be communicated throughout the world. Thus, when viewed from a disaster medicine perspective, we assert that the real issue is not variability in the frequency of rapid-onset natural disasters. Instead, it is our ability to cope adequately with the negative human health outcomes associated with those hazards.

Not surprisingly, given the kinetic energy released by rapid-onset natural disasters, such events typically cause a variety of traumatic injuries due to overpressure, penetrating wounds, and crushing including mild, moderate, and severe traumatic brain injury (TBI) to individuals in the spatial domain impacted by a natural disaster. In fact, neurotrauma comprises a major risk factor for mortality and morbidity outcomes that are directly attributable to rapid-onset natural disasters. TBI, in particular, is characterized by a complex pathophysiological process that originates with an acute primary injury that is likely to evolve over time resulting in a variety of chronic secondary injuries (6–9). In most cases, TBIs are classified based on severity and mechanism. Severity is estimated to be mild, moderate, or severe using the Glasgow coma scale (GCS) to approximate the level of consciousness based on motor, verbal, and eye responses. The injury mechanism is categorized as either closed or penetrating based on damage to the skull and meninges that surround the central nervous system (CNS) (10). Therefore, early diagnosis and treatment is a priority in all TBI incidents (11). To help place the human scale of this problem in context, the centers for disease control and prevention (CDC) estimates there approximately 1.7 million new cases per year occur in the US civilian population posing unique challenges for emergency medicine and long-term care (12).

While early diagnosis and treatment of TBI can be challenging under normal circumstances, evidence from the field reveals that those challenges are exacerbated by the chaotic environments created in the aftermath of major natural disasters. As a result, in this paper, we identify three major challenges associated with the diagnosis, treatment, and prevention of TBI in populations affected by rapid-onset natural disasters: (1) immediate emergency medical response, (2) long-term care, and (3) prevention of post-event increases in pediatric TBIs because of abuse when rapid-onset natural disasters occur.

## Immediate Emergency Care

The immediate diagnosis and treatment of rapid-onset natural disaster victims with head injuries is essential to minimize the development of secondary brain injuries. This is the premier challenge associated with delivery of emergency medical care when that category of natural disasters happens, especially in developing countries with limited medical infrastructure capacity that may be further degraded by the disaster. With some exceptions, outside the more economically developed countries, the availability of state-of-the-art neurological care is scarce. This reality is highlighted in the event of a major rapid-onset natural disaster where it is assured that neurosurgeons initially within the impact zone will be overwhelmed during a mega mass casualty event because of the surge in injured persons requiring specialized care. For example, in Indonesia, there are approximately 140 neurosurgeons for 250 million people (13). To place this in context, there are 3,500 neurosurgeons in the US for a population of 299 million (14). Thus, the importance of addressing the issue of efficient delivery of specialized medical care to meet the immediate needs of disaster victims, especially in developing countries that have a limited number of medical specialists, cannot be overstated.

Moreover, immediate emergency medical response to TBI induced by rapid-onset natural disasters, in particular severe TBIs which have a heightened potential to cause mortality, raises questions of medical ethics with respect to allocating scarce neurotrauma care resources to treat victims presenting with severe TBI who may not survive even with treatment given the circumstances as well as the absolute capacity of patient treatment capability relative to the magnitude of the disaster. Incidents such as the 8.7 magnitude earthquake that struck Indonesia on 28 March 2005 and the 7.3 magnitude earthquake in Haiti on 12 January 2010 graphically illustrate multiple emergency medicine challenges that happen simultaneously during major natural disasters. They include care of patients with complex compound fractures, spinal cord or brain injuries, burns, and other massive trauma as well as victims requiring amputation. And, because many injured people who were unfamiliar with modern Western medicine refused to be treated for serious conditions that eventually led to their predictable deaths (15), rapid-onset natural disaster victims in developing countries may benefit from a culturally sensitive outreach program that focuses on informing the public in those countries about emergency medical practice for the purposes of building trust to facilitate immediate care delivery.

We note that a computer system was developed to deliver telemetric care to rapid-onset disaster victims with TBIs in order to fill the knowledge gap for immediate emergency care created by the limited neurosurgical resources in Indonesia (13). We argue that these types of telemedicine systems have the potential to significantly increase access to a wide variety of medical resources in an equally diverse range of emergency scenarios (e.g., rapid-onset natural disasters, droughts, armed conflict, and terrorism) particularly until assets from beyond the impact zone arrive on scene. Further, we note that telemetric medicine has the greatest impact when it is used to deliver highly specialized care that may otherwise be unavailable. We also advocate the development of mobile computer software systems that improve the timeliness and quality of all types of emergency medicine.

We acknowledge that the cost associated with the development and maintenance of these types of computer systems is likely to be prohibitively high in economically disadvantaged regions where disasters are more likely to have a devastating impact. Therefore, we advocate selective investment by the international community in supporting the capital costs associated with the creation of basic indigenous capacity. This would make it possible for telemetric medicine to be leveraged during a crisis for immediate emergency care and also allow it to be utilized as a resource for long-term care. Bilateral foreign aid programs such as the activities conducted by the office of foreign disaster assistance (OFDA) within the US Agency for International Development are designed to reduce disaster risk by building capacity and helping communities become resilient to future crises. International organizations like the United Nations office for the coordination of humanitarian affairs (OCHA) have a similar mission. As a result, they can play a leading role in transferring telemetric medicine technologies to improve disaster response efforts.

## Long-Term Care

A tornado event in the US and an earthquake in the People’s Republic of China (PRC), respectively provide insights into the challenge of providing long-term care for TBI patients in the aftermath of rapid-onset natural disasters. The multiple tornadoes on 3 May 1999 in Oklahoma directly resulted in 40 deaths and 519 injured persons. Of the 519 injuries, 134 required hospitalization and 376 were treated and released. Brain injuries accounted for 39% of the hospitalizations, 12% of the patients who were treated and released, and 43% of the deaths (16). The 2008 earthquake in Sichuan caused multiple injuries in its 1,871 victims. Of the 3,177 injuries that were diagnosed in those victims, 727 (22.9%) were head injuries. Contusions accounted for 49.9% of the head injuries (11.4% of all injuries) (17, 18). We offer both of the above examples because tornadoes are common seasonal events in parts of the US while earthquakes are similarly common hazards in seismically active regions of the PRC. Moreover, those types of rapid-onset natural disasters occur in a number of other countries underscoring the generic need for long-term TBI care in the aftermath of natural disasters.

Although many individuals who suffer severe TBI due to a rapid-onset disaster do not survive, the occurrence of mild and moderate TBIs among natural disaster survivors who do not receive otherwise fatal co-morbidities leads to an increase in local demand for neurological specialists who are capable of delivering long-term care for these types of non-lethal injuries. This demand is likely to persist across the lifespan of the local TBI population, especially if the affected region experiences multiple disasters. The tornado and earthquake examples we provide certainly apply in this instance. Major tornado outbreaks occur every year in the case of Oklahoma with large numbers of injuries and deaths occurring approximately once every 10 years. Thus, Oklahoma and the other states in what commonly is referred to as the “Tornado Alley” region of the US should expect that the number of TBI patients will steadily increase because they have growing or stable populations, resulting in an increased need for specialty and general medical care and nursing in the long-term. Similarly, major areas of China are seismically active and experience intense earthquakes that cause extensive damage. In such instances, we expect that cascading populations of new patients will result in similar increases in the demand for specialty medical care. In addition to an increase in the need for neurological care, the requirement for long-term care is likely to increase demand for general medical and nursing care too including mental health services.

## Prevention of Post-Disaster Pediatric TBI

The third challenge that we elucidate is particularly vexing because it involves children who survive rapid-onset natural disasters. The incidence of inflicted and non-inflicted childhood TBI is known to increase dramatically in regions that are severely affected by natural disasters such earthquakes, floods, hurricanes, and tornadoes. To illustrate this challenge, we summarize several US studies that help place it in perspective. As reported in Ref. (19), a study was conducted to determine if post-disaster stress had an effect on the incidence of child TBI. The study focused on pediatric TBIs that resulted in the child’s admission to an intensive care unit or death near the time and location of Hurricane Floyd (NC, USA, September 1999). Statistical analysis determined that the incidence of both inflicted and non-inflicted child TBIs dramatically increased in the 6-month period immediately after the hurricane. The incidence rate ratios of inflicted and non-inflicted TBIs among children in the most severely affected counties were estimated to be 5.1 and 10.7, respectively. The increase in non-inflicted TBI was attributed to increases in the risk associated with driving, environmental hazards, lodging displacement, and a hypothesized decrease in the quality of adult supervision (19, 20). In a similar study, child abuse was observed to increase following Hurricane Hugo and the Loma Prieta Earthquake but no changes were found in abuse patterns following Hurricane Andrew (21).

This raises the question of why increased child abuse was reported in some rapid-onset natural disasters but not others. Frequently experiencing a particular type of event and having familiarity with coping with its consequences, including the possibility of increased domestic violence in its aftermath, may be one plausible explanation for the difference reported by researchers studying child abuse rates after hurricanes and earthquakes. In fact, a study conducted by the Louisiana Department of Social Services supports such an inference (21).

Taken as a group, these studies demonstrate the heightened risk of inflicted pediatric TBI following the social dislocation associated with rapid-onset natural disasters. Consequently, the problem of increased child abuse and, by extension, pediatric TBI is a vexing social issue that must be addressed by the medical and public health communities as well as the justice system. Determining which solutions are appropriate and likely to produce the best results within diverse cultures in the aftermath of a rapid-onset natural disaster is a challenging social and research question. We note that mental health services, family counseling, financial assistance, and stress management programs appear to be among viable candidate approaches. However, the *true* implications of these possible interventions in terms of preventing post-disaster pediatric TBI from exacting its toll on helpless children are largely unknown and require systematic identification to ensure eradication of this problem.

## Conflict of Interest Statement

The authors declare that the research was conducted in the absence of any commercial or financial relationships that could be construed as a potential conflict of interest.
